# Effects of Diabetes and Voluntary Exercise on IgA Concentration and Polymeric Immunoglobulin Receptor Expression in the Submandibular Gland of Rats

**DOI:** 10.3390/medicina59040789

**Published:** 2023-04-18

**Authors:** Jaebum Park, Yuko Yamamoto, Kouki Hidaka, Satoko Wada-Takahashi, Shun-suke Takahashi, Toshiya Morozumi, Nobuhisa Kubota, Makiko Saita, Juri Saruta, Wakako Sakaguchi, Masahiro To, Tomoko Shimizu, Yuko Mikuni-Takagaki, Keiichi Tsukinoki

**Affiliations:** 1 Department of Environmental Pathology, Kanagawa Dental University, 82 Inaoka, Yokosuka 2388580, Kanagawa, Japan; drparkjb@gmail.com (J.P.); sakaguchi@kdu.ac.jp (W.S.); 2 Department of Dental Hygiene, Kanagawa Dental University, Junior College, 82 Inaoka, Yokosuka 2388580, Kanagawa, Japan; yamamoto.yuko@kdu.ac.jp; 3 Department of Restorative Dentistry, Kanagawa Dental University, 82 Inaoka, Yokosuka 2388580, Kanagawa, Japan; hidaka@kdu.ac.jp; 4 Department of Oral Physiology, Kanagawa Dental University, 82 Inaoka, Yokosuka 2388580, Kanagawa, Japan; s.takahashi@kdu.ac.jp; 5 Department of Pharmacology, Kanagawa Dental University, 82 Inaoka, Yokosuka 2388580, Kanagawa, Japan; takahashi.shunsuke@kdu.ac.jp; 6 Department of Endodontics, The Nippon Dental University School of Life Dentistry at Niigata, 1-8 Hamaura-cho, Chuo-ku, Niigata 9518580, Niigata, Japan; morozumi@ngt.ndu.ac.jp; 7 Department of Diagnostic Pathology, Kanagawa Dental University, 82 Inaoka, Yokosuka 2388580, Kanagawa, Japan; n.kubota@kdu.ac.jp; 8 Department of Fixed Prosthodontics, Kanagawa Dental University, 82 Inaoka, Yokosuka 2388580, Kanagawa, Japan; saita@kdu.ac.jp; 9 Department of Education Planning, Kanagawa Dental University, 82 Inaoka, Yokosuka 2388580, Kanagawa, Japan; saruta@kdu.ac.jp; 10 Department of Clinical Oral Anatomy, Kanagawa Dental University, 82 Inaoka, Yokosuka 2388580, Kanagawa, Japan; m.tou@kdu.ac.jp; 11 Department of Implantology and Periodontology, Kanagawa Dental University, 3-31-6 Tsuruya, Kanagawa-ku, Yokohama 2210835, Kanagawa, Japan; shimizu@kdu.ac.jp; 12 Kanagawa Dental University, 82 Inaoka, Yokosuka 2388580, Kanagawa, Japan; takagaki@kdu.ac.jp

**Keywords:** saliva, immunoglobulin (Ig) A, polymeric immunoglobulin receptor (poly-IgR), diabetes, voluntary exercise, Otsuka Long–Evans Tokushima Fatty (OLETF) rat

## Abstract

*Background and Objectives*: Patients with diabetes are more susceptible to upper respiratory tract infections (URTIs) because they are easily infected. Salivary IgA (sali-IgA) levels play a major role in transmitting URTIs. Sali-IgA levels are determined by salivary gland IgA production and polymeric immunoglobulin receptor (poly-IgR) expression. However, it is unknown whether salivary gland IgA production and poly-IgR expression are decreased in patients with diabetes. While exercise is reported to increase or decrease the sali-IgA levels, it is unclear how exercise affects the salivary glands of patients with diabetes. This study aimed to determine the effects of diabetes and voluntary exercise on IgA production and poly-IgR expression in the salivary glands of diabetic rats. *Materials and Methods*: Ten spontaneously diabetic Otsuka Long–Evans Tokushima Fatty (OLETF) rats (eight-week-old) were divided into two groups of five rats each: a non-exercise group (OLETF-C) and a voluntary wheel-running group (OLETF-E). Five Long–Evans Tokushima Otsuka (LETO) rats without diabetes were bred under the same conditions as the OLETF-C. Sixteen weeks after the study began, the submandibular glands (SGs) were collected and analyzed for IgA and poly-IgR expression levels. *Results*: IgA concentrations and poly-IgR expression levels in SGs were lower in OLETF-C and OLETF-E than in LETO (*p* < 0.05). These values did not differ between the OLETF-C and OLETF-E. *Conclusions*: Diabetes decreases IgA production and poly-IgR expression in the salivary glands of rats. Moreover, voluntary exercise increases sali-IgA levels but does not increase IgA production and poly-IgR expression in the salivary glands of diabetic rats. Increasing IgA production and poly-IgR expression in the salivary glands, which is reduced in diabetes, might require slightly higher-intensity exercise than voluntary exercise under the supervision of a doctor.

## 1. Introduction

Currently, the number of people with diabetes is increasing worldwide [1]. Diabetes is associated with many complications [2]. In diabetes, chronic elevation of blood glucose levels causes damage to blood vessels, triggering major complications, such as retinopathy, kidney disease, nerve damage, myocardial infarction, and stroke [3]. In addition to these, susceptibility to infection is also another complication [4]. Diabetes is an immunodeficiency condition caused by persistently high blood glucose levels [5]. Patients with diabetes are more susceptible to upper respiratory tract infections (URTIs) because they are easily infected [6]. Moreover, patients with diabetes have a higher probability of progressing from URTIs to severe pneumonia and, consequently, have a higher risk of death [7]. Therefore, it is very important to prevent URTIs in patients with diabetes.

Prevention of URTIs is effectively achieved by increasing the antimicrobial resistance of the oral cavity, which is the entry point for foreign microorganisms [8]. Saliva is responsible for the oral cavity’s antimicrobial potential [9]. Saliva contains many antimicrobial substances, including immunoglobulin (Ig) A, IgG, IgM, lactoferrin, lysozyme, peroxidase, cystatin, and mucins [10]. IgA plays the most important role in defense against infection by pathogenic microorganisms [11]. IgA in saliva blocks pathogenic microorganisms before they infect epithelial cells of the pharynx and other respiratory organs [12]. In fact, low salivary IgA (sali-IgA) levels increase the risk of URTIs [13]. The mechanism of IgA secretion into saliva is as follows [14]. First, salivary gland plasma cells produce J-chain-mediated dimeric IgA (dIgA). dIgA then binds to the polymeric immunoglobulin receptor (poly-IgR) expressed on the basolateral surface of salivary gland acinar cells and is transported to the luminal side. Subsequently, a portion of poly-IgR detaches to form the secretory component (SC), and the complex of dIgA and SC is secreted into the saliva as secretory IgA. Therefore, sali-IgA levels are influenced by IgA production and poly-IgR expression in the salivary glands [15].

We hypothesized that patients with diabetes susceptible to URTIs might have decreased IgA production and poly-IgR expression in their salivary glands. Low sali-IgA levels have been reported in these patients [16]. However, it is unclear whether IgA production or poly-IgR expression is reduced in the salivary glands of patients with diabetes. Exercise is related to an increase or decrease in sali-IgA levels. Prolonged or strenuous exercise decreases sali-IgA levels [17,18]. In contrast, low-impact exercise, such as walking for 16 weeks, has been reported to increase sali-IgA levels [19]. Moderate exercise boosts overall immunity and increases resistance to infection [20]. Furthermore, moderate physical activity is associated with increased sali-IgA levels and a decreased risk of URTIs [20]. In our previous study with rats, voluntary exercise with a running wheel also increased IgA concentration and poly-IgR expression in salivary glands [21]. However, the effect of exercise on IgA production and poly-IgR expression in the salivary glands of patients with diabetes remains unclear. IgA production and poly-IgR expression in salivary glands are influenced by the autonomic nervous system and immune status [22]. Light-impact exercise balances the sympathetic and parasympathetic nervous systems in humans [23]. However, the effect of voluntary exercise, such as light running, on the autonomic nervous system in patients with diabetes has not been determined.

The aim of this study was to determine the effects of diabetes and voluntary exercise on IgA production and poly-IgR expression in the salivary glands of diabetic rats. In addition, the effects of voluntary exercise on the autonomic nervous system of diabetic rats were examined.

## 2. Materials and Methods

### 2.1. Animals

For the present study, 10 male Otsuka Long–Evans Tokushima Fatty (OLETF) rats (7 weeks old) and five male Long–Evans Tokushima Otsuka (LETO) rats (controls for OLETF rats; 7 weeks old) were obtained from Japan SLC, Inc. (Shizuoka, Japan). This rat strain was selected because it is commonly used in animal experiments on prediabetes and diabetes [24]. The experimental protocol used in this study was approved by the Kanagawa Dental University Ethics Committee for Animals (approval number: 17-016, issued to Y.M.-T. on 7 June 2017), and the animals were maintained in accordance with Kanagawa Dental University guidelines for the care and use of laboratory animals. All rats were maintained at a temperature of 22 ± 3 °C and kept in a 12 h light/dark cycle. The rats were checked daily for health problems. No signs of pain or distress were observed before or during the study. The rats had free access to a commercial pellet diet (MF; Oriental Yeast Co. Ltd. Tokyo, Japan) and water. After 7 days of prior habituation, the 10 OLETF rats were randomly divided into two groups of five rats each: a non-exercise group (OLETF control; OLETF-C) and a voluntary wheel-running group (OLETF exercise; OLETF-E). OLETF-C rats were maintained individually in a wire mesh cage (400 × 130 × 150 mm; Clea Japan, Tokyo, Japan) for 16 weeks. OLETF-E rats were maintained one by one in a wire mesh cage with a running wheel of 370 mm in diameter; the size of the cage was similar to that of the OLETF-C cage (Clea Japan). The OLETF-E rats were allowed to exercise voluntarily during the experimental period. Five LETO rats were maintained under the same conditions as OLETF-C rats. The rats had free access to food and water during the test period. The duration of the experiment was 16 weeks. On average, the OLETF-E rats ran 455 km in 16 weeks. The body weight of each rat was measured once a week.

### 2.2. Sampling

At the end of the 16-week experimental period, all rats were sacrificed via decapitation between 20:00 and 24:00 h after anesthetization with sevoflurane and collection of blood samples from the abdominal aorta. Cecal digesta (c-digesta), cecal tissue (c-tissue), and submandibular glands (SGs) were collected after euthanasia. The right SG samples were immediately immersed in 4% paraformaldehyde phosphate buffer solution. Other samples were weighed and stored at −80 °C until analysis. Blood samples were collected in test tubes and promptly centrifuged at 1200× *g* for 20 min at 20 °C to separate the serum, which was then stored at −80 °C until analysis.

### 2.3. Measurement of Blood Pressure and Heart Rate

Blood pressure and heart rate were measured 15 weeks later, from 10:00 to 12:00 and 14:00 to 16:00. Blood pressure and heart rate in rats were measured simultaneously at the tail vein using the tail-cuff method with Softron BP-98A (Softron Co., Ltd., Tokyo, Japan). At that time, the rat bodies were kept warm at 39–40 °C, the blood pressure and heart rate were each measured three times, and the average values were calculated.

### 2.4. Measurement of Blood Glucose Concentration

Six hours after the last diet intake, blood was drawn from the tail vein of the rats on the last day of the experimental period. Blood glucose concentration was measured using a glucose meter (FreeStyle freedom Lite^®^; Abbott Diabetes Care Inc., Alameda, CA, USA).

### 2.5. Measurement of Blood Albumin Concentration

Serum albumin concentration was measured using the improved bromocresol purple (BCP) method (Aqua-auto Kainos ALB Test Kit; KAINOS Laboratories, Inc., Tokyo, Japan) in accordance with the manufacturer’s instructions.

### 2.6. Measurement of IgA Concentration

The total IgA concentrations in the c-digesta, serum, and SGs were measured using the Rat IgA ELISA Quantitation Kit (BETHYL Laboratories Inc., Montgomery, TX, USA) according to the manufacturer’s instructions. Briefly, c-digesta samples were treated with 20 volumes of distilled water with 1 mM phenylmethylsulphonyl fluoride from dimethyl sulphoxide-dissolved stock for 60 min at 20 °C. The samples were then centrifuged (10,000× *g* for 15 min at 4 °C), and the supernatant fractions were used for IgA measurement. SG samples were crushed using a Cryo-press (Microtec Company Limited, Chiba, Japan) and mixed in PBS (0·01 M, pH 7.2–7.4; FUJIFILUM Wako Pure Chemical Corporation, Osaka, Japan) containing 1% Triton^®^ X-100 (MP Biomedicals LLC, Irvine, CA, USA) and 1 mM phenylmethylsulphonyl fluoride (from dimethyl sulphoxide-dissolved stock). These solutions were centrifuged (10,000× *g* for 15 min at 4 °C). The supernatant fraction was collected and used for enzyme-linked immunosorbent assay (ELISA).

### 2.7. Measurement of Serum IgG Concentration

The IgG concentration in the serum was measured using a Rat IgG ELISA Kit (Abcam plc, Cambridge, UK) according to the manufacturer’s instructions.

### 2.8. Measurement of Tyrosine Hydroxylase (TyrH) Concentration in SGs

The concentration of TyrH in the SGs was measured using the Rat TH ELISA Kit (Elabscience Biotechnology Co., Ltd., Houston, TX, USA) according to the manufacturer’s instructions.

### 2.9. Quantitative Real-Time Polymerase Chain Reaction (PCR) Analysis

Total ribonucleic acid (RNA) isolation from the SGs and quantitative real-time PCR analysis were performed as previously described [21]. The expression level of poly-IgR was measured via real-time PCR using the Step One Plus Real-Time PCR system (Applied Biosystems, Waltham, MA, USA). Glyceraldehyde-3-phosphate dehydrogenase (*Gapdh*) was used as the endogenous control. The sequences of the specific oligonucleotide primer pairs used in this study are listed in Supplementary Table S1. Relative gene expression was analyzed using the 2^−∆∆Ct^ method.

### 2.10. SG Tissue Preparation for Hematoxylin–Eosin Staining

SG immersed in a 4% paraformaldehyde phosphate buffer solution was stained with hematoxylin–eosin using the same procedure as in a previous study [25].

### 2.11. Statistical Analysis

Statistical analyses were performed using JMP (Version-12; SAS Institute Inc., Cary, NC, USA). All results were expressed as the mean ± standard error (SE). All statistical analyses were conducted using one-way analysis of variance (ANOVA) followed by Tukey’s post-hoc test to assess differences among groups, except for the analysis of correlations between data. The Pearson product–moment correlation coefficient was used to analyze the correlation between data. Statistical significance was set at *p* < 0.05.

## 3. Results

### 3.1. Body Weight Gain and SG Weight

**Figure 1:**
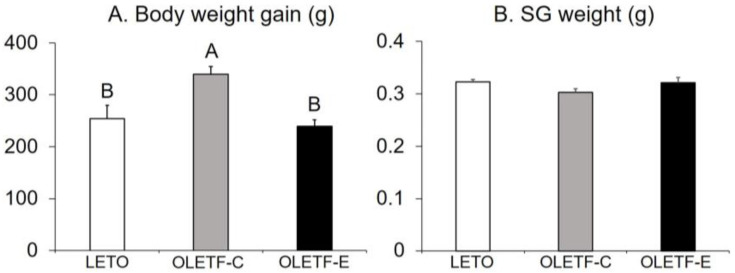
Body weight gain (**A**) and submandibular gland (SG) weight (**B**) 16 weeks after the start of this study. *n* = 5 per group. Bars represent mean values, and error bars represent the SE. Signific ant differences were determined using the one-way ANOVA, followed by multiple comparisons using Tukey’s post-hoc test. The letters represent a significant difference in body weight gain between different groups (*p* < 0.05, Tukey’s post-hoc test). There were no differences in the SG weight among the three groups (*p* = 0.2, one-way ANOVA).

There were group differences in body weight gain after 16 weeks (*p* < 0.0001, one-way ANOVA; Figure 1A). Higher values were found for OLETF-C than for LETO and OLETF-E (*p* < 0.05, Tukey’s post-hoc test; Figure 1A). No significant difference was observed between LETO and OLETF-E (Figure 1A). The SG weight did not differ among the three groups (*p* = 0.2, one-way ANOVA; Figure 1B).

### 3.2. Blood Glucose Concentration, Mean Blood Pressure, Serum Albumin Concentration, and Heart Rate

**Figure 2:**
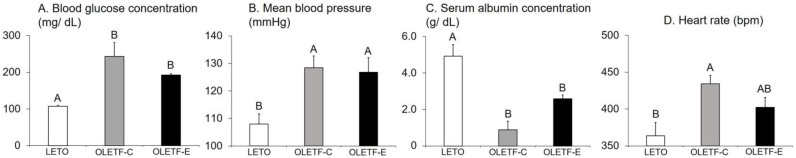
Blood glucose concentration (**A**) and serum albumin concentration (**C**) 16 weeks after the start of this study. Blood glucose levels were measured 6 h after eating. Mean blood pressure (**B**) and heart rate (**D**) 15 weeks after the start of this study. *n* = 5 per group. The bars represent mean values, and the error bars represent SE. Significant differences were determined using the one-way ANOVA, followed by multiple comparisons using Tukey’s post-hoc test. The letters represent significant differences between groups (*p* < 0.05, Tukey’s post-hoc test).

Blood glucose concentrations and mean blood pressure (estimated using average systolic and diastolic blood pressure) significantly differed among the groups (*p* = 0.003 and *p* = 0.01, respectively, one-way ANOVA; Figure 2A,B), with OLETF-C and OLETF-E exhibiting higher values than LETO (*p* < 0.05, Tukey’s post-hoc test; Figure 2A,B), and no significant difference was observed between OLETF-C and OLETF-E (Figure 2A,B). Serum albumin concentrations also significantly differed among the groups (*p* = 0.0002, one-way ANOVA; Figure 2C), with OLETF-C and OLETF-E exhibiting lower values than LETO (*p* < 0.05, Tukey’s post-hoc test; Figure 2C). No significant difference was observed between OLETF-C and OLETF-E (Figure 2C). The groups also exhibited differences in heart rate (*p* = 0.02, one-way ANOVA; Figure 2D). Higher values were found for OLETF-C than for LETO (*p* < 0.05, Tukey’s post-hoc test; Figure 2D). No significant differences were found between LETO and OLETF-E or between OLETF-C and OLETF-E (Figure 2D).

### 3.3. Histological Changes in SGs due to Diabetes Mellitus and Voluntary Exercise

**Figure 3:**
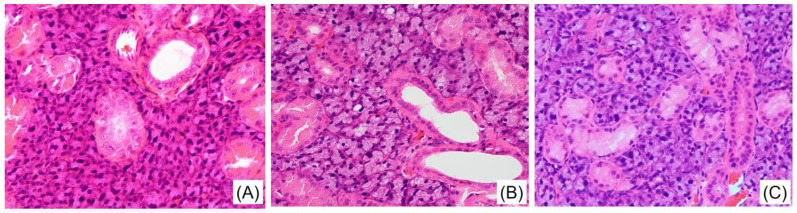
Hematoxylin–eosin staining of the submandibular gland (SG) tissue 16 weeks after the start of this study in the LETO (**A**), OLETF-C (**B**), and OLETF-E (**C**) groups. Magnification (400×).

Histological analysis of the SG using hematoxylin–eosin staining showed no significant differences among the three groups. In all three groups, there was no change in the status of cell atrophy, hypertrophy, fenestration, or inflammatory cell infiltration (Figure 3).

### 3.4. c-Tissue Weight, c-Digesta Weight, and IgA Concentrations in c-Digesta

**Figure 4:**
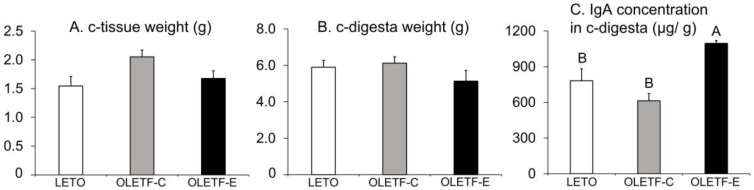
Cecal tissue (c-tissue) weight (**A**), cecal digesta (c-digesta) weight (**B**), and IgA concentrations in c-digesta (**C**) 16 weeks after the start of this study. *n* = 5 per group. The bars represent mean values, and the error bars represent SE. Significant differences were determined using the one-way ANOVA, followed by multiple comparisons using Tukey’s post-hoc test. The letters represent significant differences between groups (*p* < 0.05, Tukey’s post-hoc test). There were no significant differences in the c-tissue and c-digesta weights among the three groups (*p* = 0.06 and 0.3, one-way ANOVA).

The c-tissue and c-digesta weights did not significantly differ among the three groups (*p* = 0.06 and *p* = 0.3, respectively, one-way ANOVA; Figure 4A,B). There were group differences in IgA concentrations in the c-digesta (*p* = 0.001, one-way ANOVA; Figure 4C), with OLETF-E exhibiting higher values than LETO and OLETF-C (*p* < 0.05, Tukey’s post-hoc test; Figure 4C). No significant difference was observed between LETO and OLETF-C (Figure 4C).

### 3.5. IgA and IgG Concentrations in Serum

**Figure 5:**
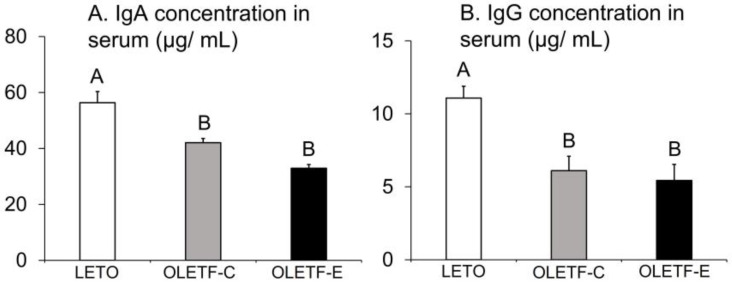
IgA concentration (**A**) and IgG concentration (**B**) in the serum 16 weeks after the start of this study. *n* = 5 per group. The bars represent mean values, and the error bars represent SE. Significant differences were determined using the one-way ANOVA, followed by multiple comparisons using Tukey’s post-hoc test. The letters represent significant differences between groups (*p* < 0.05, Tukey’s post-hoc test).

Serum IgA and IgG concentrations significantly differed among the groups (*p* = 0.0001 and *p* = 0.003, respectively, one-way ANOVA; Figure 5A,B), with lower values found in OLETF-C and OLETF-E than in LETO (*p* < 0.05, Tukey’s post-hoc test; Figure 5A,B). No significant difference was observed between OLETF-C and OLETF-E (Figure 5A,B).

### 3.6. IgA Concentration, Poly-IgR mRNA Expression Level, and TyrH Concentration in SGs

**Figure 6:**
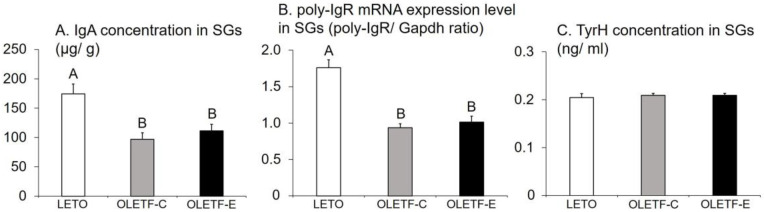
IgA concentrations (**A**), poly-IgR mRNA expression level (**B**), and TyrH concentration (**C**) in the submandibular glands (SGs) 16 weeks after the start of this study. *n* = 5 per group. The bars represent mean values, and the error bars represent SE. Significant differences were determined using the one-way ANOVA, followed by multiple comparisons using Tukey’s post-hoc test. The letters represent significant differences between groups (*p* < 0.05, Tukey’s post-hoc test).

The groups significantly differed in terms of IgA concentrations and poly-IgR mRNA expression levels in SGs (*p* = 0.004 and *p* < 0.0001, respectively, one-way ANOVA; Figure 6A,B), with lower values in OLETF-C and OLETF-E than in LETO (*p* < 0.05, Tukey’s post-hoc test; Figure 6A,B), and no significant difference was observed between OLETF-C and OLETF-E (Figure 6A,B). In contrast, the TyrH concentration in the SGs did not differ significantly among the three groups (*p* = 0.8, one-way ANOVA; Figure 6C).

### 3.7. Correlations of IgA Concentration in the SGs with Different Parameters

The IgA concentration in the SGs had a significant positive correlation with the poly-IgR mRNA expression level in the SGs (r = 0.76, *p* = 0.001), serum IgA concentration (r = 0.68, *p* = 0.005), serum albumin concentration (r = 0.63, *p* = 0.01), and serum IgG concentration (r = 0.60, *p* = 0.02) and a significant negative correlation with heart rate (r = −0.76, *p* = 0.001), mean blood pressure (r = −0.62, *p* = 0.01), and blood glucose concentration (r = −0.59, *p* = 0.02), based on Pearson’s correlation coefficients (*n* = 15 for all items; Table 1). The IgA concentration in the SGs was not significantly correlated with the c-digesta weight (r = 0.16, *p* = 0.6), IgA concentration in the c-digesta (r = 0.056, *p* = 0.8), c-tissue weight (r = −0.44, *p* = 0.1), or TyrH concentration in the SGs (r = −0.11, *p* = 0.7), based on Pearson’s correlation coefficients (*n* = 15 for all items; Table 1).

### 3.8. Correlation of Poly-IgR mRNA Expression Level in the SGs with Different Parameters

The poly-IgR mRNA expression level in the SGs had a significant positive correlation with serum IgA concentration (r = 0.81, *p* = 0.0002), serum albumin concentration (r = 0.73, *p* = 0.002), and serum IgG concentration (r = 0.64, *p* = 0.01) and a significant negative correlated with heart rate (r = −0.75, *p* = 0.001), mean blood pressure (r = −0.64, *p* = 0.01), and blood glucose concentration (r = −0.64, *p* = 0.01), based on Pearson’s correlation coefficients (*n* = 15 for all items; Table 2). In contrast, the poly-IgR mRNA expression level in the SGs was not significantly correlated with the IgA concentration in the c-digesta (r = 0.094, *p* = 0.7), c-digesta weight (r = 0.051, *p* = 0.9), c-tissue weight (r = −0.37, *p* = 0.2), and TyrH concentration in the SGs (r = −0.10, *p* = 0.7), based on Pearson’s correlation coefficients (*n* = 15 for all items; Table 2).

## 4. Discussion

In the present study, we examined the effects of diabetes and voluntary exercise on IgA production and poly-IgR expression in the salivary glands. In this study, the OLETF-C and OLETF-E groups had higher blood glucose concentrations, higher mean blood pressures, higher heart rates, and lower serum albumin concentrations than the LETO group. These conditions are observed in diabetes [26,27,28]. Based on these results, the OLETF rats used in this study were diabetic. Several human studies reported decreased sali-IgA levels in patients with diabetes [16,29]. In fact, when the sali-IgA flow rate is low, salivary gland IgA concentrations are also low [30], as are the expression levels of poly-IgR in the salivary glands [30,31]. Although sali-IgA levels could not be measured in this experiment, IgA concentration and poly-IgR expression in the SG represented sali-IgA levels. Moderate exercise has positive effects in alleviating diabetes, such as improved blood glucose control [32]. In addition, several studies reported that moderate exercise increases sali-IgA levels [19,33]. Based on these reports, we hypothesized that IgA levels and poly-IgR expression in the salivary glands are decreased in diabetic rats but that both may be increased by voluntary exercise using a running wheel. In Japan, from 2010 to 2016, the mortality rate of adults with pneumonia was high at 27% [34]. Furthermore, 30% of patients with pneumonia had diabetes [34]. If this experiment can determine how diabetes affects sali-IgA levels and how to increase them, it could contribute to developing preventive measures for pneumonia in patients with diabetes.

IgA concentration and poly-IgR expression in the SGs were associated with serum albumin concentration, heart rate, blood pressure, and blood glucose levels, indicative of diabetes pathology. This indicates that diabetes is associated with IgA production and poly-IgR expression in the SGs. However, histological analysis of the SG showed no differences among the three groups. Therefore, diabetes did not affect the SG tissue.

IgA concentrations in the SGs of the OLETF-C and OLETF-E groups were lower than those in the SGs of the LETO group. Furthermore, poly-IgR mRNA expression in the SGs was reduced in the diabetic groups. Autonomic nerve stimulation influences IgA production and poly-IgR expression in salivary glands. Proctor et al. reported that increased sympathetic and parasympathetic stimulation increased IgA production and poly-IgR mRNA expression in the salivary glands [22]. Furthermore, IgA secretion into saliva was increased by both parasympathetic and sympathetic stimulation [35]. In addition, decreased sympathetic and parasympathetic stimulation was shown to decrease sali-IgA secretion and salivary gland poly-IgR expression [22,35]. Therefore, sympathetic and parasympathetic activation might have been reduced in the diabetic rat groups. Elevated mean blood pressure and heart rate are markers of sympathetic activation [36]; in the present study, the mean blood pressure and heart rate of the diabetic group were higher than those of the control group. In addition, TyrH concentrations, a measure of local sympathetic activation [37], did not differ between the diabetic and control groups in the SG tissue. Furthermore, no association was found between TyrH concentration in the SGs and IgA concentration in the SGs or poly-IgR expression levels in the SGs. Therefore, the sympathetic nerve activity of diabetic rats was not reduced in the present study. Since the mean blood pressure, heart rate, and TyrH concentration in the SGs were not positively correlated with IgA concentration or poly-IgR mRNA expression levels in the SGs, the level of sympathetic activation would not have been related to lower IgA concentration or poly-IgR mRNA expression levels in the SGs. Autonomic nervous system disturbances have been reported as complications of diabetes [38,39]. Furthermore, parasympathetic activity is significantly suppressed in patients with diabetes [40], and decreased parasympathetic activity occurs within a short period after the onset of diabetes [41]. The 24-week-old OLETF rats were in the early diabetic period [42]. Thus, in the present study, early diabetes reduced parasympathetic stimulation and decreased IgA and poly-IgR expressions in the SG.

Serum IgA and IgG concentrations in the OLETF-C and OLETF-E groups were lower than those in the LETO group. In addition, IgA and poly-IgR expression levels in the SGs were associated with serum IgA and IgG levels. Serum Ig levels represent a state of humoral immunity [43]. In fact, patients with pneumonia who had compromised humoral immunity and higher severity scores had lower serum IgG and IgA concentrations than those who did not [44]. People with diabetes are known to have reduced humoral immunity [45]. Sali-IgA concentration is related to humoral immunity, and it decreases with decreased humoral immune activity, in which plasma cells produce Ig via CD4^+^ T helper cells [46]. Humoral immunity is also involved in poly-IgR expression [47]. In mice with autoimmune disease, the serum IgG level is high, but upon improvement, the serum IgG is low, and salivary gland poly-IgR expression is also reduced [47]. Based on these reports, in the present study, diabetes caused a reduction in humoral immunity that lowered serum IgG and IgA levels, which, in turn, reduced IgA production and poly-IgR expression in the SG.

No differences in c-tissue and c-digesta weights were observed among the three groups. However, IgA concentrations in the c-digesta were higher in the OLETF-E group than in the LETO and OLETF-C groups. Liu et al. reported that exercise did not alter the weight of rat c-digesta [48], and the same result was obtained in this experiment. Exercise was shown to alter the gut microbiota and affect immunity [49]. Moderate exercise was shown to increase IgA levels in the mouse ileum [50]. The levels of IgA^+^ plasma cells; interleukin-2, -4, -6, and -10; transforming growth factor-β; interferon-γ; and tumor necrosis factor were increased in the mouse ileum of the moderate exercise group, compared to those in the no exercise group [50]. Similarly, in the present study, voluntary exercise also increased IgA^+^ plasma cells and inflammatory cytokines in the rat intestine, resulting in an increase in IgA concentration in the c-digesta. In this experiment, no correlation was found between IgA concentrations in the c-digesta and IgA concentrations and poly-IgR expression in the SGs. Thus, the effects of voluntary exercise, such as increasing IgA concentrations in the c-digesta, were not associated with SG IgA production and poly-IgR expression.

No differences in IgA concentrations or poly-IgR expression in the SGs were observed between OLETF-C and OLETF-E. In our previous study using rats, voluntary exercise with running wheels resulted in high IgA concentrations, poly-IgR expression in the SG, and sali-IgA flow rates [21]. In the present study, IgA concentration and poly-IgR expression in the SGs were lower in the OLETF group of diabetic rats than in the LETO group. The rats in the OLETF group performed voluntary exercise on the same running wheels as in our previous study; however, IgA levels and poly-IgR expression in the SGs did not increase. Human studies reported that moderate exercise with a slight load, but not low-strength exercise, increased sali-IgA levels [20,51]. The increase in sali-IgA levels with regular and moderate exercise was attributed to the CD4^+^ T helper cells as a factor contributing to the increased sali-IgA levels. [20]. CD4^+^ T helper cell counts were also decreased in patients with diabetes [52]. Low-impact voluntary exercise did not increase low CD4^+^ T helper cell counts in patients with diabetes. In these patients with low levels of IgA production in the salivary glands, slightly strenuous exercise, rather than voluntary exercise, increased IgA production in the SG. Light-impact exercise balances the sympathetic and parasympathetic nervous systems [23]. However, it might not be effective in patients with diabetes and autonomic imbalance. Thus, voluntary exercise might not have increased IgA production or poly-IgR expression in the SG of diabetic rats. Future experiments are needed to examine these parameters at two or three different levels of exercise intensity.

This was the first study to determine the effects of diabetes and voluntary exercise on IgA production and poly-IgR expression in the salivary glands; this study was significant but had some limitations. First, our experimental method did not allow us to evaluate IgA concentrations in the SG and sali-IgA levels in a single experiment [30]. Therefore, it was not possible to collect saliva from rats and analyze sali-IgA levels. Sali-IgA concentration and poly-IgR expression represented the sali-IgA levels [30,31]. Thus, the sali-IgA secretion rate was lower in the OLETF diabetic rat model in this study. Second, since the purpose of this study was to examine the effect of voluntary exercise on IgA production and poly-IgR expression in the SG of diabetic rats, the exercise intensity of the rats was set to that which we reported with healthy nondiabetic rats [21]. Future experiments are needed to determine the effect of exercise intensity on sali-IgA levels and salivary gland poly-IgR expression in diabetic as well as normal control rats. Third, we did not establish an exercise group for the nondiabetic LETO group. This was because our previous study found that in nondiabetic rats, voluntary exercise using the same running wheel as in the present experiment increased IgA concentration and poly-IgR expression levels in the SG [21]. In future experiments, more accurate results will be obtained if an exercise group is set up for the nondiabetic rats.

## 5. Conclusions

Diabetes resulted in decreased IgA production and poly-IgR expression in rat salivary glands. Voluntary exercise increased sali-IgA levels in nondiabetic rats but did not increase IgA production and poly-IgR expression in the salivary glands of diabetic rats. Increasing IgA production and poly-IgR expression in the salivary glands, which is reduced in diabetes, might require slightly higher-intensity exercise than voluntary exercise under the supervision of a doctor.

## Figures and Tables

**Table 1:** Correlation between immunoglobulin (Ig) A concentrations in the submandibular glands (SGs) and different parameters.

	IgA Concentrations in SGs		
	r *	*p*	*n*
poly-IgR ^§^ mRNA expression level in SGs	0.76	0.001	15
IgA concentration in serum	0.68	0.005	15
Albumin concentration in serum	0.63	0.01	15
IgG concentration in serum	0.60	0.02	15
c-digesta ^‡^ weight	0.16	0.6	15
IgA concentration in c-digesta	0.056	0.8	15
Heart rate	−0.76	0.001	15
Mean blood pressure	−0.62	0.01	15
Blood glucose concentration	−0.59	0.02	15
c-tissue ^#^ weight	−0.44	0.1	15
TyrH ^†^ concentration in SGs	−0.11	0.7	15

poly-IgR ^§^: polymeric immunoglobulin receptor. c-digesta ^‡^: cecal digesta. c-tissue ^#^: cecal tissue. TyrH ^†^: tyrosine hydroxylase. * Pearson’s correlation coefficient.

**Table 2:** Correlation between polymeric immunoglobulin receptor (poly-IgR) expression levels in the submandibular gland (SG) and different parameters.

	Poly-IgR mRNA Expression Levels in SGs		
	r *	*p*	*n*
IgA concentration in serum	0.81	0.0002	15
Albumin concentration in serum	0.73	0.002	15
IgG concentration in serum	0.64	0.01	15
IgA concentration in c-digesta	0.094	0.7	15
c-digesta ^‡^ weight	0.051	0.9	15
Heart rate	−0.75	0.001	15
Mean blood pressure	−0.64	0.01	15
Blood glucose concentration	−0.64	0.01	15
c-tissue ^#^ weight	−0.37	0.2	15
TyrH ^†^ concentration in SGs	−0.10	0.7	15

c-digesta ^‡^: cecal digesta. c-tissue ^#^: cecal tissue. TyrH ^†^: tyrosine hydroxylase. * Pearson’s correlation coefficient.

## Data Availability

Data are available upon request to the corresponding author.

## Supplementary Materials

The following supporting information can be downloaded at: https://www.mdpi.com/article/10.3390/medicina59040789/s1, Table S1: Primers used for analysis of gene expression in the submandibular gland (SG).
