# Supplementary material for: Effects of Diabetes and Voluntary Exercise on IgA Concentration and Polymeric Immunoglobulin Receptor Expression in the Submandibular Gland of Rats
Source: Medicina (Kaunas). 2023 Apr 18;59(4):789. doi: 10.3390/medicina59040789 (PMC10144866; doi:10.3390/medicina59040789)
Supplement: Supplementary file 1 [file medicina-59-00789-s001.zip › Supplemental Table S1.pdf]

Supplemental Table S1. Primers used for analysis of gene expression in the submandibular gland (SG)

| | Primer sequence (5’-3’) | |
|---|---|---|
| Target gene | Forward | Reverse |
| poli-IgR* | CAGTCCTCGAAGGAAAAGATGAAAT | CAGGAATGCTGAGTAGGCCA |
| *Gapdh* | GTATCGGACGCCTGGTTAC | CTTGCCGTGGGTAGAGTCAT |

poli-IgR*: polymeric immunoglobulin receptor
